# Meeting report: advancing practical applications of biodiversity ontologies

**DOI:** 10.1186/1944-3277-9-17

**Published:** 2014-12-08

**Authors:** Ramona L Walls, Robert Guralnick, John Deck, Adam Buntzman, Pier Luigi Buttigieg, Neil Davies, Michael W Denslow, Rachel E Gallery, J Jacob Parnell, David Osumi-Sutherland, Robert J Robbins, Philippe Rocca-Serra, John Wieczorek, Jie Zheng

**Affiliations:** 1 The iPlant Collaborative, University of Arizona, Tucson, AZ, USA; 2 University of Colorado, Boulder, CO, USA; 3 University of California at Berkeley, Berkeley, CA, USA; 4 Department of Immunobiology, University of Arizona, Tucson, AZ, USA; 5 Alfred-Wegener-Institut Helmholtz Zentrum für Polar- und Meeresforschung, MARUM Center for Marine Sciences, University of Bremen, Bremen, Germany; 6 Gump South Pacific Research Station, University of California Berkeley, Moorea, French Polynesia; 7 National Ecological Observatory Network, Boulder, CO, USA; 8 School of Natural Resources and the Environment, University of Arizona, Tucson, AZ, USA; 9 European Bioinformatics Institute, Wellcome Genome Campus, Cambridge, UK; 10 University of California at San Diego, La Jolla, CA, USA; 11 University of Oxford e-Research Centre, Oxford, UK; 12 Department of Genetics and Institute of Biomedical Informatics, Perelman School of Medicine, University of Pennsylvania, Philadelphia, PA, USA

**Keywords:** Ontology, Biodiversity, Population, Community, Darwin Core, OWL, RDF, Microbial ecology, Sequencing

## Abstract

We describe the outcomes of three recent workshops aimed at advancing development of the Biological Collections Ontology (BCO), the Population and Community Ontology (PCO), and tools to annotate data using those and other ontologies. The first workshop gathered use cases to help grow the PCO, agreed upon a format for modeling challenging concepts such as ecological niche, and developed ontology design patterns for defining collections of organisms and population-level phenotypes. The second focused on mapping datasets to ontology terms and converting them to Resource Description Framework (RDF), using the BCO. To follow-up, a BCO hackathon was held concurrently with the 16^th^ Genomics Standards Consortium Meeting, during which we converted additional datasets to RDF, developed a Material Sample Core for the Global Biodiversity Information Framework, created a Web Ontology Language (OWL) file for importing Darwin Core classes and properties into BCO, and developed a workflow for converting biodiversity data among formats.

## Introduction

Biological data range from information about small-scale material entities such molecules and cells to large-scale processes such as ecosystem carbon fluxes. Formal description of this complexity is needed to create semantically rich data that can enable efficient aggregation, querying, and ultimately machine reasoning. The imperative for this is growing as data pipelines open wider with new technologies such as high-throughput sequencing and remote sensing. Standards development has been a critical endeavor to reconnect scattered and poorly described biodiversity data [1,2], and new W3C standard semantic approaches utilizing the ontology language OWL2 [3] and Resource Description Framework (RDF) [4], offer richer ways to express relationships among data and query the results.

The biodiversity informatics and standards community is embracing these new approaches and developing two key new ontologies: the Biological Collections Ontology (BCO) and the Population and Community Ontology (PCO). For more background on these ontologies, see [5]. The BCO focuses on how to model material samples and observations, while the PCO models assemblages of individuals and their interactions. Large-scale studies being undertaken by scientists across the globe, such as the wide range of physical specimens and environmental data collected by the National Ecological Observatory Network (NEON) [6], have provided strong use cases to guide the development of these ontologies. We report here on development workshops for the PCO and BCO that were held from 18–20 Feb 2014 at the iPlant Collaborative in the Bio5 Institute of the University of Arizona in Tucson, Arizona, USA, as well as a BCO hackathon held in conjunction with the Genomics Standards Consortium (GSC) meeting from 31 March–2 April 2014 at Pembroke College in Oxford, UK. Workshop and hackathon participants are listed in Table 1.

**Table 1:** Participants at all events

**Name (Affiliation)**	**PCO workshop**	**BCO workshop**	**BCO hackathon**
Kyle Braak (Global Biodiversity Information Framework)			x
Matthew Brush (Oregon Heath & Science University)^A^	x		
Adam Buntzman (University of Arizona)		x	
Pier Buttigieg (Alfred-Wegener-Institut, Bremerhaven/MARUM, Bremen)	x	x	
Neil Davies (Gump Station, Moorea, University of California)	x	x	
John Deck (Berkeley Natural History Museum, University of California)^B^	x	x	x
Michael Denslow (National Ecological Observatory Network)		x	
Melissa Haendel (Oregon Heath & Science University)^A^	x		
Bonnie Hurwitz (University of Arizona)		x	
Jim Hu (Texas A&M University)	x	x	
Rachel Gallery (University of Arizona)		x	
Robert Guralnick (University of Colorado)^B^	x	x	x
Fiona McCarthy (University of Arizona	x		
Peter Midford	x	x	
Norman Morrison (BioVel)		x	
David Osumi Sutherland (European Bioinformatics Institute)^A^	x		
Jacob Parnell (National Ecological Observatory Network)		x	
Robert Robbins (University of California San Diego)		x	x
Philippe Rocca-Serra (University of Oxford e-Research Centre)	x	x	x
Mike Trizna (Smithsonian Institution)			x
Ramona Walls (iPlant Collaborative, University of Arizona)^B^	x	x	x
John Wieczorek (Berkeley Natural History Museum, University of California)		x	x
Pelin Yilmaz (Max Plank Institute, Bremen)^A^		x	
Jie Zheng (University of Pennsylvania)	x	x	

^A^Remote participant.

^B^Organizer.

## PCO workshop

The goal of the PCO workshop was to better specify this newly-emerging ontology by: 1) producing a set of use cases specific to the PCO; 2) determining if existing PCO terminology was adequate for those use cases, and, if not, develop a candidate list of new terms to meet their needs; 3) proposing several methods for annotating data on population level phenotypes to be presented for further discussion at the Phenotype Research Coordination Network [7] annual meeting that followed the workshop.

Prior to the workshop, participants compiled a list of use cases and suggested terms. These were discussed and augmented at the meeting. Out of the use cases came a discussion of the different types of collections of organisms described in biological studies and different ways of defining them. Rather than commit to a specific definition of the broad and heterogeneous concept of population, we decided that the best strategy was to first identify the fundamental characteristics by which groups of organisms (i.e. ‘populations’ in the loose sense) were commonly defined. Based on those characteristics, we then can define classes for groups of organisms as needed. Table 2 lists some characteristics discussed at the workshop that will be incorporated into PCO. We note that some groupings define social constructs and that these can apply to human groups as well as other organisms.

**Table 2:** Some of the fundamental ways that organisms can be grouped into collections

**Characteristic**	**Examples**
Embedded in the same system(s)	• Proximity in time and space to nuclear blast
	• Bird arrival times to summer breeding grounds
Engaging in the same processes	• Non-feeding nutrient exchange
	• Coordinated behaviors in a social setting (e.g., calling behaviors in response to predators)
	• Interactions (host-symbiont; competition)
Sharing common descent	• Genetic similarity
	• Member of a genetically connected population
	• Phylogenetic relationships
Sharing similar morphology, physiology, behavior (whether by descent or convergence)	• Flight
	• Fossorial locomotor pattern
	• Countercurrent respiration
	• Quorum sensing in bacteria
Socially constructed characteristics	• Membership in an organization
	• Legal status
	• Education level

Using characteristics like those in Table 2, we composed a simple ontology design pattern (ODP) to define collections of organisms. This ODP can be generalized as follows:

*collection of organisms with characteristicX*^a^ = def:

object aggregate

and **has_member** only (*organism* and **relation** some *characteristicX*)

and **has_member** min 2 *organism*

where *object aggregate*[8] comes from the BFO [9,10]. This could be translated into a textual definition such as: “A *collection of organisms with characteristicX* is an *object aggregate* that has as members a minimum of two *organisms* with the *characteristicX* and only *organisms* with the *characteristic X*”.

Membership of an individual organism in some collection is defined in the PCO using general class axioms such as:

*organism* and (**relation** some *characteristicX*)

SubClassOf **member of** some *collection of organisms with characteristicX*

meaning (in plain text): “Any *organism* that has some *characteristicX* is a member of the class of *organisms with characteristicX*.”

We use this and similar patterns within the PCO to automatically classify collections of organisms using standard OWL reasoning software, and such reasoning can furthermore be applied to triple stores of instance data. In practice, collections of organisms are defined using specific characteristics such as having a quality, participating in a process, or bearing a role. These patterns provide simple examples utilizing the core PCO vocabulary; defining most biologically interesting groups of organisms will generally require multiple differentiae. Furthermore, defining the exact nature of many groups of organisms (e.g., those based on an interaction or a process) will rely on additional inferences derived from the logical definitions of processes such as *mutualism* or *predation*. Such definitions represent a next step for the PCO.

### Specific use cases for PCO

Two breakout groups were formed to investigate specific use cases for the PCO. The first focused on using the PCO and related ontologies to describe a longitudinal study on malaria. In this study, 100 households were selected from three different locations, each household having one or more person(s) living in the same structure and sharing meals or lodgings. Over a two-year period, blood samples were collected from enrolled study subjects and used for various assays (e.g., parasite detection and genetic analysis of host and parasite). From this use case, the new ontology term *household* was proposed and added to the PCO.

The second breakout group tackled the challenging concept of ecological niche and how it could be represented in PCO and/or the Environment Ontology (ENVO) [11]. Ecological niche occupies a unique and important place in ecological theory. Different ecologists have formulated niches in different ways [12-14], some more focused on spatial ecological meaning, some in community ecological frames, and yet others related directly to species physiological tolerances. Previous work in ENVO had conceptualized the niche as an environment that would allow a given species to maintain and expand its population. Ultimately, the group decided against attempting to create a single ontology class, which is unlikely to satisfy all camps. Rather, the group favored efforts directed at creating classes (for example, as subclasses of *environmental condition*[15] that, together with the use of other ontology terms and instance-level data (e.g., physicochemical parameters and spatial coordinates) would allow scientists to define the niche plastically. These characteristics or conditions could be used to query datasets, in order to determine which organisms satisfy the criteria of a given niche concept or habitat suitability range, rather than forcing scientist to match their data to pre-modeled classes in any ontology. For specific uses, equivalent classes for niches can be constructed within application ontologies.

### Population level phenotypes

There are many ways that phenotypes can be modeled using an ontology [16-19], and the choice of a model will depend on the use case. Furthermore, there are many phenotypic qualities that can be measured either at the individual, population or even the species level, such as plant height, hair color, or presence of a limb. Of main concern for the PCO are phenotypes that are relevant only at the level of a population. We divide these into two main classes:

1. Those that are statistics describing an aggregate of all of the individual phenotypes in the population, such as population reproductive rate (the sum of individual reproductive rates) or penetrance (the proportion of individuals carrying a particular genetic variant that also express an associated phenotype),

2. Those that can only exist in a population as a whole, such as population growth rate or group behaviors such as flocking or schooling.

Workshop participants decided that it made sense for the PCO to provide pre-composed terms for population-level phenotypes that can exist only in populations, but not to pre-compose population-level phenotypes that are simple aggregate functions of individual phenotypes; these belong in taxon specific trait ontologies instead. For example, the Drosophila Phenotype ontology is using PCO terms and design patterns to record how mortality rates vary among populations [20].

### PCO workshop outcomes

• Preliminary list of factors by which organisms are grouped into populations or communities.

• Ontology design pattern for how to describe membership in a group of organisms.

• New PCO terms for specific use cases.

• Decisions about modeling challenging concepts such as ecological niche that span PCO and ENVO.

• Decision to provide pre-composed terms for those characteristics of populations that cannot be defined as derived from individual measurements.

## BCO workshop and hackathon

Work at the Oxford hackathon built directly on Tucson workshop, therefore we report on the outcomes of both events together in the following sections. The goals of the BCO workshop in Tucson were to: 1) coordinate development with the Ontology for Biomedical Investigations (OBI); 2) collect several biodiversity datasets and annotate them with BCO and other ontology terms; 3) load data into a triple store and run test queries in SPARQL Protocol and RDF Query Language (SPARQL); 4) identify subsets of the Darwin Core (DwC) vocabulary to represent as either classes or properties of BCO. The follow-up hackathon in Oxford brought together a smaller group to: 1) further model tabular spreadsheets using BCO terms; 2) develop necessary workflow components to convert such data into formats that promote different uses (e.g., interchange, archiving, publication); 3) complete coding of relevant DwC terms within BCO; 4) adapt Darwin Core Archive publishing mechanisms to deliver a material sample ‘core’. These innovations are discussed below.

For participants who were new to ontologies, the first part of the Tucson workshop was spent introducing ontologies in general, as well as specifics of the BCO, OBI, and ENVO. There were also demonstrations of some tools that can be used for working with ontologies such as ISATools [21] for mapping data structures to ontology terms and Protégé [22] for creating and viewing ontologies. The Biocode-FIMS [23] was introduced and used during the workshop to convert the test datasets to instances represented in RDF/XML and coded with BCO terms.

### Mapping Darwin Core terms to ontologies

Darwin Core (DwC) [1,24] is a set of standards for exchanging biodiversity data that includes a glossary of approximately 200 terms and definitions. The DwC vocabulary has been formally described in RDF [25] in order to facilitate its re-use, but it intentionally has a very limited class-property hierarchical structure. Other than a handful of organizing classes, all terms in the DwC vocabulary are properties (i.e. sub-properties of [26]). In contrast, the majority of terms in BCO are classes (subclasses of [27]).

Participants prioritized a list of DwC terms [28] based on how often they appear in datasets aggregated by the Global Biodiversity Information Facility (GBIF) [29]. Beginning with commonly used DwC terms, the group discussed how the terms would be defined in an ontological framework and noted any existing ontology terms that corresponded to concepts in the DwC. For example, the Darwin Core property ‘sex’ was found to correspond to the term *biological sex*[30] from the Phenotypic Quality Ontology or PATO. The majority of DwC terms could not be mapped readily to existing ontologies. For these, a preliminary step was made to describe the DwC terms in natural language closely modeling the semantics that will eventually be realized when appropriate terms are added to the BCO.

During the Oxford hackathon, an ontology called “dwcterms.owl” was created that translates DwC as RDF into OWL, with DwC classes interpreted as ontology classes and DwC properties interpreted as datatype properties. There are 17 classes in DwC as RDF, ten in the “dwctype” namespace and seven in the “terms” namespace, but only 15 of the classes are unique, as *Occurrence* and *Taxon* occur in both namespaces. Specification of mappings between classes in dwcterms.owl and bco.owl is ongoing, and work in progress can be viewed in the most current version of the BCO [31]. One hundred fifty one datatype properties were imported into BCO as part of dwcterms.owl, including two properties (**bibliographicCitation** and **date**) that are included only as organization properties and should not be used in annotation. As described in the preceding paragraph, work is ongoing to determine which of these should remain as data properties in the BCO and which should be modeled as object properties or classes.

### Annotating biodiversity datasets with ontologies

Testing the usefulness of the BCO for its intended purposes requires running queries over actual data. We gathered several datasets that were or could be marked up with metadata from DwC and MIxS. During the course of the workshop and hackathon, we were able to map four datasets to ontology terms: a hypothetical DNA marker gene (barcoding) dataset based on a composite of several real datasets, a soil microbial dataset from the NEON, a DwC taxonomic archive of centipedes, and an Ocean Sampling Day (OSD) [32] dataset. Mappings were done by first discussing the meaning of spreadsheet column headings and assigning ontology classes to particular columns or sets of columns on an annotated sheet. When this was completed, we used the Biocode-FIMS tool to convert spreadsheet data to RDF. As an example of this process, we describe some of the mappings built for the NEON and OSD datasets.

#### NEON dataset

NEON data was the output of a survey in which soils were sampled at various locations. A number of genetic and physio-chemical parameters (e.g., DNA marker gene, pH, Ca concentration, temperature) were analyzed for each soil sample. During the workshop, we realized that DNA marker gene assays were not adequately modeled in OBI, so a breakout group was formed to develop a model, which was later compared to an existing ISA-Tab mapping. Details of the ontological model and how it fits into OBI and ISA-Tab will be described in a separate publication presented at the 2014 International Conference on Biomedical Ontologies.

The NEON dataset was converted to RDF/XML, coded using a combination of OBI, BCO, and DwC terms. A globally unique identifier (GUID) was created for each instance by attaching a globally unique and valid URI prefix to a locally unique column value. For example, the “NEON_sample_name” column value was used to create the GUID for instances of the class of *specimen*[33] that represent soil core source samples. In cases where a locally unique identifier was not expressed on the spreadsheet, an identifier was constructed by digesting values from a range of cells that represented that instance.

**Figure 1:**
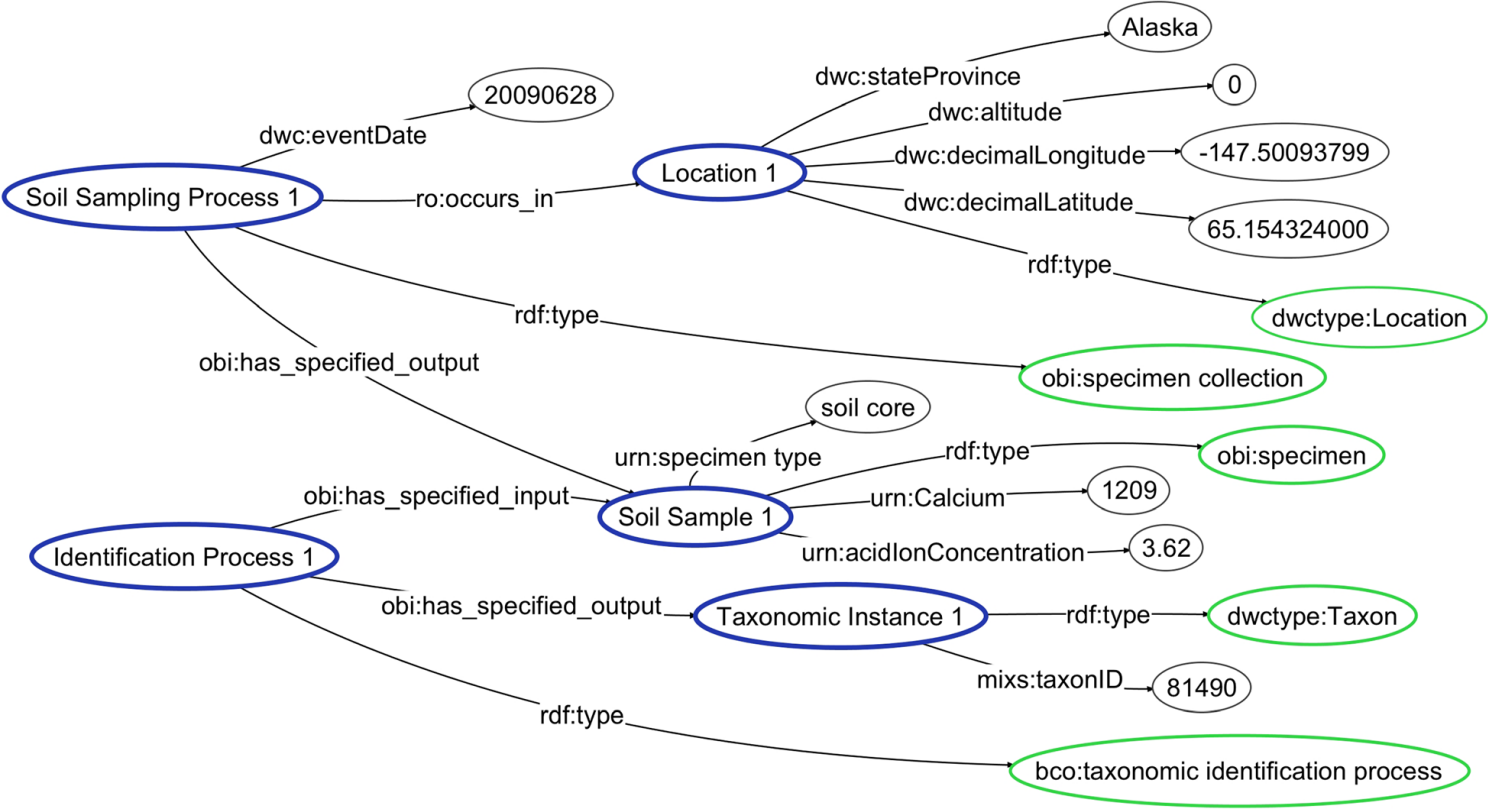
**NEON dataset.** A directed graph representing of a subset of the NEON soil sampling data that was coded into RDF, according to the semantics of the Biological Collections Ontology (BCO). In this graph, an instance of the type obi:specimen collection called “Soil Sampling Process 1” occurs in “Location 1”, which is an instance of the class dwctype: Location. Blue ovals represent instances, green ovals ontology classes, and black ovals literals, whereas relations (a.k.a. properties) are represented as arrows. Properties and classes are prefixed with an abbreviation from the vocabulary from which they come (ro = Relations Ontology, bco = Biological Collections Ontology, obi = Ontology for Biomedical Investigations, dwc = Darwin Core, dwctype = DwC Type Vocabulary, mixs = MIxS as RDF), except for properties that do not yet exist in a controlled vocabulary and are prefixed with “urn”. Labels have been altered for readability from the original data set used to generate this graph.

After all class and instance-level entities were created, relationships were expressed using relations from the Relation Ontology (RO) and OBI, based on the class-level semantic structure of the BCO. For example, based on the BCO axiom *specimen collection*[34]**has_specified_output**[35]*specimen*[33], we expressed that each instance of the process of *specimen collection* (representing NEON’s soil sampling processes) had as specified output an instance of *specimen* (representing NEON’s soil cores). All outputs of the mappings were stored as an RDF/XML file (available at [36]), and a graphical representation of a part of the dataset mapped to BCO is shown in Figure 1.

A sample SPARQL query was constructed against the resulting RDF to filter on pH values associated with soil cores resulting from sampling processes:

PREFIX xsd: <http://www.w3.org/2001/XMLSchema#>

select * from < urn:uuid:f5baecb4-6b83-4716-99f9-5e3ebf22ead2 > where

{

?materialSamplingProcess < obi:has_specified_output > ?soilCore .

?materialSamplingProcess a < obi:materialSamplingProcess > .

?soilCore < obi:acidIonConcentration > ?ph .

filter(xsd:decimal(?ph) <3.7)

}

#### OSD dataset

**Figure 2:**
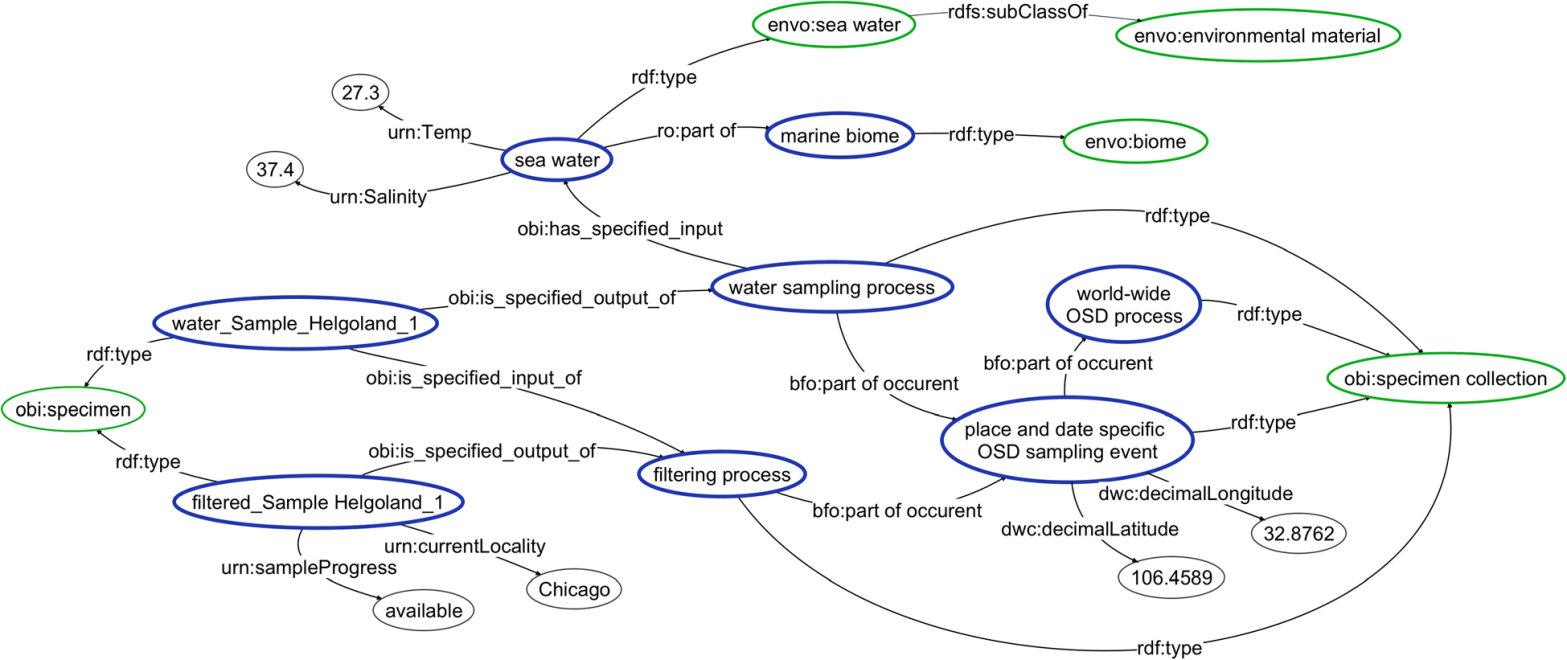
**OSD dataset.** A directed graph representing a subset of the Ocean Sampling Day prototype data that was coded into RDF according to the semantics of the Biological Collections Ontology (BCO). In this graph, there are two instances of the type obi:specimen called “water_Sample_Helgoland1” and “filtered_Sample_Helgoland1) linked by an instance of obi:specimen collection called “filtering process”. Blue ovals represent instances, green ovals ontology classes, and black ovals literals, whereas relations (a.k.a. properties) are represented as arrows. Properties and classes are prefixed with an abbreviation from the vocabulary from which they come (ro = Relations Ontology, envo = Environment Ontology, obi = Ontology for Biomedical Investigations, dwc = Darwin Core, bfo = Basic Formal Ontology,), except for properties that do not yet exist in a controlled vocabulary and are prefixed with “urn”. Labels have been altered for readability from the original data set used to generate this graph.

Ocean Sampling Day [32] is an event that occurred in 2014 on the same day in many locations across the globe, particularly sites participating in the Genomic Observatories Network [37]. Ocean water is collected at a specified location and time and filtered to extract a range of microorganisms. Pertinent environmental parameters (such as salinity and temperature) are measured and sample sites are annotated with *biome*[38] and *environmental feature*[39] classes. Material from the filter is ultimately sequenced to provide data on microbial ecology and diversity. A dataset representing samples collected at one of the pilot sampling events for OSD was mapped to BCO using the same procedure we used for the NEON data. Instances of classes were recorded in an RDF/XML document (available at [40]). A diagram showing some of the OSD data coded with ontology terms is shown in Figure 2.

### Data formats and conversion tools

**Figure 3:**
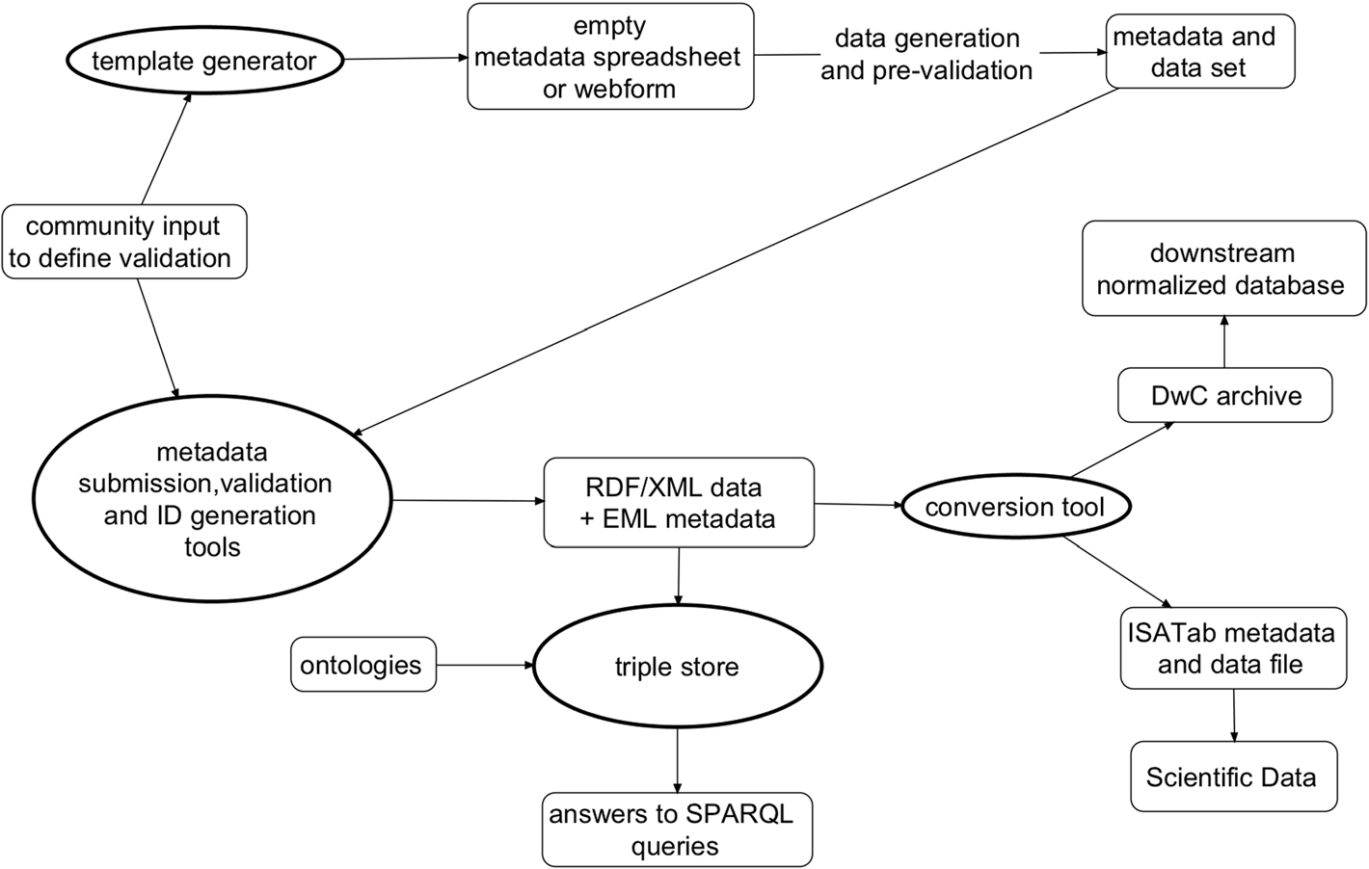
**Workflow for semantic data conversion.** Workflow showing how spreadsheets used during field collecting can be brought into a linked open data context, with conversions from core RDF/XML data and EML metadata formats into tools for further discovery (e.g. Darwin Core Archives) or for archiving and publishing (ISA-Tab). Such conversions are not “lossless” but they do move the data sources to well-established and needed consumers or publication venues such as Scientific Data. Answers to scientific queries require the additional input of ontologies. Rounded rectangles represent information content entities and ovals represent conversion tools.

Development of tools that simplify publishing of ontology-annotated data must go hand in hand with ontology development. One of the core objectives of the Oxford hackathon was to build a BCO exchange format for easy translation to Darwin Core Archives (DwCA) [41] and ISA-Tab [21]. DwCA is designed as a simple encoding standard for sharing biodiversity datasets and their metadata and is the most common mechanism used to aggregate data for large-scale initiatives such as VertNet [42] and GBIF. ISA-Tab – a format used for expressing biomedical and metagenomic data – is increasingly used for biodiversity inventories and will be the format of choice for a new journal, Scientific Data. Many datasets could be expressed in either format, for instance: biotic inventories with encoded sampling processes, DNA marker gene studies, and any biodiversity data consisting of tissue, DNA-extract, PCR, or sequence derivatives.RDF/XML is a highly expressive standard format for expressing ontologies and data that could be adopted as a transfer format for BCO-coded data. Translations between formats should begin with RDF/XML and proceed from that point to both DwCA and ISA-Tab, because DwCA and, to a lesser degree, ISA-Tab lose information during encoding. That is, it is often not possible to completely restore the information in RDF/XML by back-conversion from DwCA or ISA-Tab, because neither of those formats are capable of expressing the complex semantics contained in some RDF/XML datasets. A workflow diagram expressing how data could move between formats is shown in Figure 3. An important part of this workflow is collecting data with sufficient semantic content, thus the workflow begins with the development of a semantically-enabled template generator.

A breakout session during the last day of the Oxford hackathon developed a proof-of-concept tool that converted existing RDF outputs to DwCA, utilizing an ontology specification described in [43] and defined more fully in [44], and implementing GBIF’s DwCA reader Java library. This conversion tool enables the user to specify the input format (n-triples, n3, Turtle, RDF/XML) and which DwCA core to use (taxonomy, occurrence, material sample). The tool is represented in Figure 3 as “RDF conversion tool” and handles output to DwCA. The second half of the tool, conversion to ISA-Tab, is still in development. The code that was completed before the end of the hackathon was committed to the BiSciCol Triplifier codebase [45] and will be available soon as part of the Triplifier command-line toolkit.

### Material sample core Darwin Core Archive

The other breakout session focused on developing a much-needed Material Sample Core for DwCA, utilizing the MaterialSample class that was added to the DwC in 2013. The idea behind the Material Sample Core is remarkably simple – it will be used to cover those Darwin Core Occurrences that are based on physical material (e.g., biological specimens) that are collected rather than on observations that are reported (e.g., in a field notebook) or represented on various media (e.g., digital photographs). By using the DwC MaterialSample class to cover specimens (“living specimen”, “fossil specimen”, “preserved specimen” from the DwC type vocabulary) and physical derivations thereof, we hope to make a positive step in the semantic differentiation of the two uses for the DwC Occurrence class. In practice, the Material Sample Core and the Occurrence Core contain all of the terms of the Simple Darwin Core [28]. The only difference between the two is that the record level identifier for the Material Sample Core is dwc:materialSampleID rather than dwc:occurrenceID. For various types of specimens, both terms could be populated with the same identifier, thus minimizing the impact on data aggregators such as GBIF. For material subsamples taken from specimens, the dwc:materialSampleID for the subsamples would be distinct from their parent dwc:materialSampleID.

The Material Sample Core breakout session resulted in an update to the previous draft of the Material Sample Core [46] posted in the “Under Development” section of the GBIF Extensions [47]. We then republished a specimen record dataset by mapping record identifier in the source dataset to both dwc:materialSampleID and dwc:occurrenceID, creating the first test dataset to use the new Core format.

We believe this seemingly small change is in fact very important. By grouping a large variety of types under “Occurrence”, it was previously difficult to develop extensions that made sense for material sampling processes related to the original sample or specimens. A Material Sample Core allows specimens to be published and for extensions that can describe downstream processing steps. Crucially, it also allows for the inclusion of environmental samples (e.g., of water or soil) that contain organisms and can link to derivatives such as DNA sequences. The next steps of this process include continued development with GBIF ahead of the newest release of the Integrated Publishing Toolkit, which will allow for more flexible publishing of cores, and working with the biodiversity informatics community to establish new practices given this major change.

### BCO Tucson workshop and Oxford hackathon outcomes

• Updated version of BCO [48], including import of a new ontology called dwcterms.owl.

• Enhanced coordination with ENVO and OBI.

• Concept map for DNA marker gene studies that will lead to new terms for OBI.

• Proof-of-concept mappings of four datasets to ontologies and conversion to triple stores.

• First pass of DwC and MIxS term mappings to ontology terms.

• Workflow diagram showing the context of an exchange format specification for converting among tabular data, RDF, ISA-Tab, and DwCA.

• Proof-of-concept conversion tool that converts existing RDF outputs to DwCA, utilizing an ontology specification.

• Release of a new Material Sample Core in GBIF Integrated Publishing Toolkit.

## Conclusions and future directions

The outcomes of the three workshops described in this report represent a significant step forward in the practical application of semantic web technologies to biodiversity informatics, yet work remains before the tools described herein can be put into everyday use. Although both PCO and BCO are already available for use and are in fact being used by external projects, much work remains to be done on those ontologies. For the PCO, important next steps include logically defining processes such as predation or mutualism (in collaboration with the Gene Ontology), adding more characteristics to define populations, and pre-composing selected terms for population phenotypes. Ongoing work in the BCO includes specifying domains and ranges for DwC properties and adding corresponding classes, mapping to MIxS metadata, and coordinating with similar efforts to build biomedical biobank ontologies [49].

User-friendly tool development is a crucial next step both for the use of BCO and PCO and for biodiversity informatics in general. The tools we hope to see developed soon are a semantically-enabled template generator and a more robust version of the format conversion tool described in this paper. Additional work – particularly in outreach and education – is need to implement publication of Material Sample Cores in DwCA format, and to establish a set of good practices for collecting and preserving semantically-rich biodiversity data and metadata.

## Endnotes

^a^Hypothetical or real ontology classes are printed in italics throughout, hypothetical or real relations in bold.

## Abbreviations

BCO: Biological Collections Ontology; DwC: Darwin Core; DwCA: Darwin Core Archives; ENVO: Environment Ontology; GBIF: Global Biodiversity Information Facility; GSC: Genomics Standards Consortium; GUID: globally unique identifier; MIxS: Minimum Information for any (x) Sequence; NEON: National Ecological Observatory Network; OBI: Ontology for Biomedical Investigations; ODP: Ontology design pattern; OSD: Ocean Sampling Day; OWL: Web Ontology Language; PCO: Population and Community Ontology; RDF: Resource Description Framework; RO: Relation Ontology; SPARQL: SPARQL Protocol and RDF Query Language; W3C: World Wide Web Consortium.

## Competing interests

The authors declare that they have no competing interests.

## Authors’ contributions

All authors participated in one or more of the meetings and contributed to the writing of this document. All participants across all three meetings are listed in Table 2. All authors read and approved the final manuscript.

## References

[B1] Wieczorek J Bloom D Guralnick R Blum S Döring M Giovanni R Robertson T Vieglais D Darwin Core: an evolving community-developed biodiversity data standard PLoS One 2012 7 1 e29715 10.1371/journal.pone.0029715 22238640 PMC3253084

[B2] Yilmaz P Gilbert JA Knight R Amaral-Zettler L Karsch-Mizrachi I Cochrane G Nakamura Y Sansone S-A Glöckner F Field D The Genomic Standards Consortium: bringing standards to life for microbial ecology ISME J 2011 5 10 1565 7 10.1038/ismej.2011.39 21472015 PMC3176512

[B3] Web Ontology Language [Internet] Available from: http://www.w3.org/TR/owl2-primer/

[B4] Resource Description Format [Internet] Available from: http://www.w3.org/RDF/

[B5] Walls RL Deck J Guralnick R Baskauf S Beaman R Blum S Bowers S Buttigieg PL Davies N Endres D Gandolfo MA Hanner R Jannings A Krishtalka L Matsunage A Midford P Morrison N Tuama ÉÓ Schildhauer M Smith B Stucky B Thomer A Wieczorek J Whitacre J Wooley J Semantics in support of biodiversity knowledge discovery: an introduction to the Biological Collections Ontology and related ontologies PLoS One 2014 9 3 e89606 10.1371/journal.pone.0089606 24595056 PMC3940615

[B6] Keller M Schimel DS Hargrove WW Hoffman FM A continental strategy for the National Ecological Observatory Network Front Ecol Environ 2008 6 5 282 4 10.1890/1540-9295(2008)6[282:ACSFTN]2.0.CO;2

[B7] Phenotype RCN [Internet] Available from: http://www.phenotypercn.org/

[B8] Identifier for *object aggregate* from BFO [Internet] Available from: http://purl.obolibrary.org/obo/BFO_0000027

[B9] Grenon P Smith B SNAP and SPAN: towards dynamic spatial ontology Spat Cogn Comput 2004 4 1 69 103

[B10] Smith B On classifying material entities in Basic Formal Ontology Proceedings of the third interdisciplinary ontology meeting; Tokyo 2012 Tokyo: Keio University Press 1 13 Available from: http://abelard.flet.keio.ac.jp/ontology/index.php?InterOntology12

[B11] Buttigieg PL Morrison N Smith B Mungall CJ Lewis SE The environment ontology: contextualising biological and biomedical entities J Biomed Semant 2013 4 1 43 10.1186/2041-1480-4-43 PMC3904460 24330602

[B12] Elton CS Animal Ecology 1927 New York: Macmillan Company

[B13] Grinnell J The niche-relationships of the California thrasher Auk 1917 34 4 427 33 10.2307/4072271

[B14] Hutchinson GE Concluding remarks Cold Spring Harb Symp Quant Biol 1957 22 2 415 27

[B15] Identifier for *environmental condition* from ENVO [Internet] Available from: http://purl.obolibrary.org/obo/ENVO_01000203

[B16] Gkoutos GV Green ECJ Mallon AM Hancock JM Davidson D Using ontologies to describe mouse phenotypes Genome Biol 2005 6 1 R8 15642100 10.1186/gb-2004-6-1-r8 PMC549069

[B17] Dahdul WM Balhoff JP Engeman J Grande T Hilton EJ Kothari C Lapp H Lundberg JG Midford PE Vision TJ Westerfield M Mabee PM Evolutionary characters, phenotypes and ontologies: curating data from the systematic biology literature PLoS One 2010 5 5 e10708 10.1371/journal.pone.0010708 20505755 PMC2873956

[B18] Köhler S Doelken SC Mungall CJ Bauer S Firth HV Bailleul-Forestier I Black GCM Brown DL Brudno M Campbell J FitzPatrick DR Eppig JT Jackson AP Freson K Girdea M Helbig I Hurst JA Jähn J Jackson LG Kelly AM Ledbetter DH Mansour S Martin CL Moss C Mumford A Ouwehand WH Park S-M Riggs ER Scott RH Sisodiya S The Human Phenotype Ontology project: linking molecular biology and disease through phenotype data Nucleic Acids Res 2013 42 D1 D966 74 24217912 10.1093/nar/gkt1026 PMC3965098

[B19] Park CA Bello SM Smith CL Hu Z-L Munzenmaier DH Nigam R Smith JR Shimoyama M Eppig JT Reecy JM The Vertebrate Trait Ontology: a controlled vocabulary for the annotation of trait data across species J Biomed Semant 2013 4 1 13 10.1186/2041-1480-4-13 PMC3851175 23937709

[B20] Costa M Reeve S Grumbling G Osumi-Sutherland D The Drosophila anatomy ontology J Biomed Semant 2013 4 1 32 10.1186/2041-1480-4-32 PMC4015547 24139062

[B21] Rocca-Serra P Brandizi M Maguire E Sklyar N Taylor C Begley K Field D Harris S Hide W Hofmann O Neumann S Sterk P Tong W Sansone S-A ISA software suite: supporting standards-compliant experimental annotation and enabling curation at the community level Bioinformatics 2010 26 18 2354 6 10.1093/bioinformatics/btq415 20679334 PMC2935443

[B22] Protégé [Internet] Available from: http://protege.stanford.edu/

[B23] Biocode FIMS [Internet] Available from: https://code.google.com/p/biocode-fims/

[B24] Darwin Core [Internet] Available from: http://rs.tdwg.org/dwc/

[B25] Darwin Core as RDF [Internet] Available from: http://rs.tdwg.org/dwc/rdf/dwcterms.rdf

[B26] Identifier for *Property* from RDF [Internet] Available from: http://www.w3.org/1999/02/22-rdf-syntax-ns#Property

[B27] Identifier for *Class* from RDF [Internet] Available from: http://www.w3.org/2000/01/rdf-schema#Class

[B28] Simple Darwin Core Term List [Internet] Available from: http://rs.tdwg.org/dwc/terms/simple/index.htm

[B29] Global Biodiversity Information Facility [Internet] Available from: http://www.gbif.org/

[B30] Identifier for *biological sex* from PATO [Internet] Available from: http://purl.obolibrary.org/obo/PATO_0000047

[B31] Biological Collections Ontology [Internet] Available from: https://raw.githubusercontent.com/tucotuco/bco/master/src/ontology/bco.owl

[B32] Ocean Sampling Day [Internet] Available from: http://www.oceansamplingday.org

[B33] Identifier for *specimen* from OBI [Internet] Available from: http://purl.obolibrary.org/obo/OBI_0000747

[B34] Identifier for *specimen collection* from OBI [Internet] Available from: http://purl.obolibrary.org/obo/OBI_0000659

[B35] Identifier for has_specified_output from OBI [Internet] Available from: http://purl.obolibrary.org/obo/OBI_0000299

[B36] RDF/XML File Mapping NEON Dataset to BCO [Internet] Available from: http://tinyurl.com/omteote

[B37] Davies N Meyer C Gilbert JA Amaral-Zettler L Deck J Bicak M Rocca-Serra P Sansone S-A Willis K Field D A call for an international network of genomic observatories (GOs) GigaScience 2012 1 1 5 10.1186/2047-217X-1-5 23587188 PMC3617453

[B38] Identifier for *biome* from ENVO [Internet] Available from: http://purl.obolibrary.org/obo/ENVO_00000428

[B39] Identifier for *environmental feature* from ENVO [Internet] Available from: http://purl.obolibrary.org/obo/ENVO_00002297

[B40] RDF/XML File Mapping OSD Dataset to BCO [Internet] Available from: http://tinyurl.com/n8ghbe3

[B41] Darwin Core Archive Validator [Internet] Available from: http://tools.gbif.org/dwca-validator/

[B42] Constable H Guralnick R Wieczorek J Spencer C Peterson AT VertNet: a new model for biodiversity data sharing PLoS Biol 2010 8 2 e1000309 10.1371/journal.pbio.1000309 20169109 PMC2821892

[B43] Stucky B Deck J Conlin T Ziemba L Cellinese N Guralnick R The BiSciCol Triplifier: bringing biodiversity data to the Semantic Web BMC Bioinformatics 2014 15 257 10.1186/1471-2105-15-257 25073721 PMC4124153

[B44] BiSciCol Blog Post [Internet] Available from: http://biscicol.blogspot.com/2013/03/biscicol-triples-and-darwin-core.html

[B45] BiSciCol Triplifier [Internet] Available from: http://biscicol.org/triplifier/

[B46] Material Sample Core [Internet] Available from: http://tools.gbif.org/dwca-validator/extension.do?id=http://rs.tdwg.org/dwc/terms/MaterialSample

[B47] GBIF Extensions Under Development [Internet] Available from: http://tools.gbif.org/dwca-validator/extensions.do

[B48] 2014-05-27 Version of BCO [Internet] Available from: http://purl.obolibrary.org/obo/bco/releases/2014-05-27/bco.owl

[B49] Brochhausen M Fransson MN Kanaskar NV Eriksson M Merino-Martinez R Hall RA Norlin L Kjellqvist S Hortlund M Topaloglu U Hogan WR Litton J-E Developing a semantically rich ontology for the biobank-administration domain J Biomed Semant 2013 4 23 10.1186/2041-1480-4-23 PMC4021870 24103726

